# Pax6 Regulates the Expression of Dkk3 in Murine and Human Cell Lines, and Altered Responses to Wnt Signaling Are Shown in FlpIn-3T3 Cells Stably Expressing Either the Pax6 or the Pax6(5a) Isoform

**DOI:** 10.1371/journal.pone.0102559

**Published:** 2014-07-16

**Authors:** Siri Forsdahl, Yury Kiselev, Rune Hogseth, Janne E. Mjelle, Ingvild Mikkola

**Affiliations:** 1 Research Group of Pharmacology, Department of Pharmacy, UiT – The Artic University of Norway, Tromsoe, Norway; 2 Norwegian Translational Cancer Research Center, Department of Medical Biology, UiT – The Arctic University of Norway, Tromsoe, Norway; University of Alabama at Birmingham, United States of America

## Abstract

Pax6 is a transcription factor important for early embryo development. It is expressed in several cancer cell lines and tumors. In glioblastoma, PAX6 has been shown to function as a tumor suppressor. Dickkopf 3 (Dkk3) is well established as a tumor suppressor in several tumor types, but not much is known about the regulation of its expression. We have previously found that Pax6 and Pax6(5a) increase the expression of the *Dkk3* gene in two stably transfected mouse fibroblast cell lines. In this study the molecular mechanism behind this regulation is looked at. Western blot and reverse transcriptase quantitative PCR (RT-qPCR) confirmed higher level of *Dkk3* expression in both Pax6 and Pax6(5a) expressing cell lines compared to the control cell line. By the use of bioinformatics and electrophoretic mobility shift assay (EMSA) we have mapped a functional Pax6 binding site in the mouse *Dkk3* promoter. The minimal *Dkk3* promoter fragment required for transcriptional activation by Pax6 and Pax6(5a) was a 200 bp region just upstream of the transcriptional start site. Mutation of the evolutionary conserved binding site in this region abrogated transcriptional activation and binding of Pax6/Pax6(5a) to the mouse *Dkk3* promoter. Since the identified Pax6 binding site in this promoter is conserved, RT-qPCR and Western blot were used to look for regulation of *Dkk3/REIC* expression in human cell lines. Six of eight cell lines tested showed changes in *Dkk3/REIC* expression after *PAX6* siRNA knockdown. Interestingly, we observed that the Pax6/Pax6(5a) expressing mouse fibroblast cell lines were less responsive to canonical Wnt pathway stimulation than the control cell line when TOP/FOP activity and the levels of active β-catenin and GSK3-β Ser9 phosphorylation were measured after LiCl stimulation.

## Introduction

Pax6 belongs to the paired box family of transcription factors, and plays an important role in the development of the central nervous system, the eyes, the nose and the pancreas (reviewed in [1]). Pax6 has two DNA binding domains called the paired domain and the paired-type homeodomain [2], [3], which can also participate in protein-protein interactions [4]. The transcriptional activation domain is located C-terminally, and has been shown to be a target of the Erk1/2 and p38 MAP kinases [5]. The Pax6(5a) isoform is characterized by a 14 amino acid insertion in the N-terminal part of the paired domain. This insertion changes the DNA binding specificity of the Pax6 paired domain from the consensus primarily recognized by a single paired domain [6], to a consensus looking like two tandem repeats, preferably bound by four Pax6(5a) paired domains simultaneously [2], [7]. Both Pax6 isoforms are expressed together in various tissues in the eye and brain, and seem to interact functionally to stimulate transcription of target genes [8]. However, when stably transfected and expressed in FlpIn-3T3 cell lines, the Pax6- and Pax6(5a) isoforms are able to regulate gene expression independently, with both common and isoform specific target genes [9]. An increasing number of Pax6 target genes relevant for the development and function of the brain, eyes and pancreas have been reported over the years, both based on functional studies and gene expression microarrays. Recently, an extensive analysis with combinations of ChIP-chip and gene expression microarrays identified more than 5000 promoters and about 10 different Pax6 binding motifs occupied by PAX6 in three different tissues (forebrain, lens and pancreatic β–cells). Around 1000 of these were occupied in two tissues, and 131 in all three tissues [10]. In addition to its important role in embryo development, *Pax6* is expressed in several tumors of the brain, pancreas and eye [11]–[13]. A link between the Wnt pathway and Pax6 is reported both in the brain [14], in the lens [15] and in the cornea [16].

The Wnt signaling pathway is important for normal embryogenesis, but is also reported to participate in tissue regeneration, neurodegenerative disease development and cancer progression (reviewed in [17]). The canonical Wnt pathway involves signaling molecules binding to the frizzled receptor and the LRP5/6 co-receptors, generating a signaling cascade that ultimately leads to the stabilization of β–catenin. β-catenin translocates to the nucleus, interacts with transcription factors from the Tcf/LEF family, and activates transcription by replacing repressor complexes like Groucho (reviewed in [18]). The Dickkopf family is a family of secreted factors (Dkk1–4) that interfere with the Wnt signaling pathway. Dkk1, Dkk2 and Dkk4 function as inhibitors of the canonical Wnt pathway by binding the LPR5/6 receptors and Kremen1/2 (Krm 1/2) leading to internalization, which prevents binding and activation by Wnt [19]. Dkk3 does not intereact with Krm1, Krm2 and LRP6 on the cell surface [19], [20]. It can however interact with the Krm receptor intracellularly to promote Wnt signaling [20]. There are reports on Dkk3 both enhancing and repressing the Wnt signaling pathway [20], [21], but not much is known about the mechanism. Dkk3 is downregulated and considered to function as a tumor suppressor in a number of tumor types [22], [23]. However, for some tumors the downregulation of Dkk3 is correlated with tumor progression [24], [25]. Hypermethylation of the human *Dkk3* promoter [26] may be the mechanism for the downregulation in various tumour types, as is repression of *Dkk3* by the MYCN regulated miRNA-92 [27], [28]. In addition, *Dkk3* expression is restricted by chromatin modifications [29], [30]. Nothing is known about individual transcription factors regulating *Dkk3* expression on the promoter level.

Here we show that Pax6 and Pax6(5a) regulate the expression of Dkk3 in murine fibroblast cell lines stably transfected with either of two Pax6 isoforms. RT-qPCR and Western blot show a higher level of Dkk3 expression in both Pax6 and Pax6(5a) cell lines compared to the control cell line. By the use of bioinformatics, reporter gene assays and electrophoretic mobility shift assays we have mapped a functional Pax6 binding site in the mouse *Dkk3* promoter. This site is conserved in the human *Dkk3/REIC* promoter, and RT-qPCR and Western blot indicate that PAX6 regulates Dkk3/REIC expression in human cell lines as well. The FlpIn-3T3 Pax6 and -Pax6(5a) cell lines are less responsive to canonical Wnt pathway stimulation than the control cell line.

## Results

### *Dkk3* expression is upregulated in the FlpIn-3T3 Pax6- and Pax6(5a) cell lines and the protein is localized in cytoplasm and nucleus

**Figure 1:**
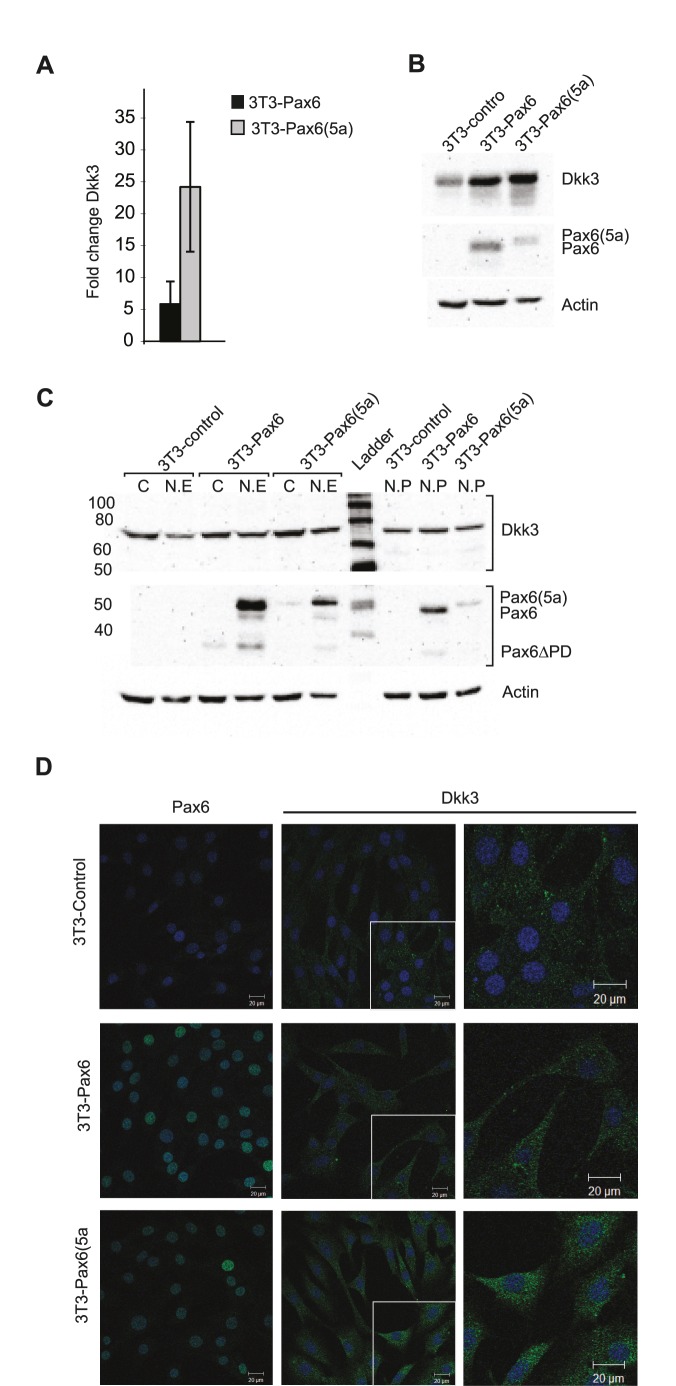
FlpIn-3T3 cells stably expressing Pax6 or Pax6(5a) have increased expression of Dkk3. A, RT-qPCR shows a 6-fold expression of *Dkk3* in the Pax6 cell line, and a 24-fold expression of *Dkk3* in the Pax6(5a) cell line, compared to the untransfected FlpIn-3T3 control cell line. B, Western blot confirms higher expression levels of Dkk3 protein in the two Pax6 containing cell lines. The observed molecular weight of Dkk3 is between 60 and 70 kD due to glycosylation. For Pax6 it is 48 kD, Pax6(5a) 50 kD and for Actin 43 kD. C, Western blot of fractionated cell extracts showing expression of Dkk3 and Pax6 (C; cytoplasm, N:E; nuclear extract, N.P; nuclear pellet). The nuclear pellet contains chromatin and remnants of the cell membrane. D, confocal imaging of immunostained FlpIn-3T3Control, -Pax6 and -Pax6(5a) cells demonstrates that the Dkk3 protein is localized in cytoplasm and nucleus. Draq5 (blue) was used to visualize the nuclei. Indicated scalebar = 20 µM.

In search for genes regulated by Pax6 we used mouse fibroblast FlpIn-3T3 cells to generate two cell lines stably expressing either the Pax6- or the Pax6(5a) isoform. Gene expression microarray identified *Dkk3* to be the gene strongest upregulated by Pax6(5a) [9]. RT-qPCR verified the strong regulation, and Pax6 ChIP confirmed direct binding of both Pax6 and Pax6(5a) to the *Dkk3* promoter in the FlpIn-3T3 cell lines [9]. As shown in Fig. 1A, *Dkk3* expression is increased 24-fold in the Pax6(5a) cell line compared to the control cell line. Also in the *Pax6* expressing cell line there is a 6-fold increase in expression of *Dkk3*. This phenomenon was also observed on Western blots (Fig. 1B). The difference between the observed (60–70 kD) and expected (38 kD) molecular weight of Dkk3 can probably be explained by N-glycosylation [37]. Depending on the cell type, Dkk3 is reported to be secreted, localized in the cytoplasm in the perinuclear region (most probably ER) or in the nucleus [38]. We made nuclear and cytoplasmic extracts and confirmed expression of Dkk3 in both compartments, in addition to the nuclear pellet (Fig. 1C). We also observed secreted Dkk3 in conditioned media from all the FlpIn-3T3 cell lines (data not shown). Immunostaining of fixed FlpIn-3T3 cell lines confirmed Dkk3 localization in the cytoplasm and nucleus of all three cell lines (Fig. 1D), with the strongest expression in the Pax6(5a) cell line, followed by the Pax6 cell line.

### *Dkk3/REIC3* expression is regulated by PAX6 in several human cell lines

**Figure 2:**
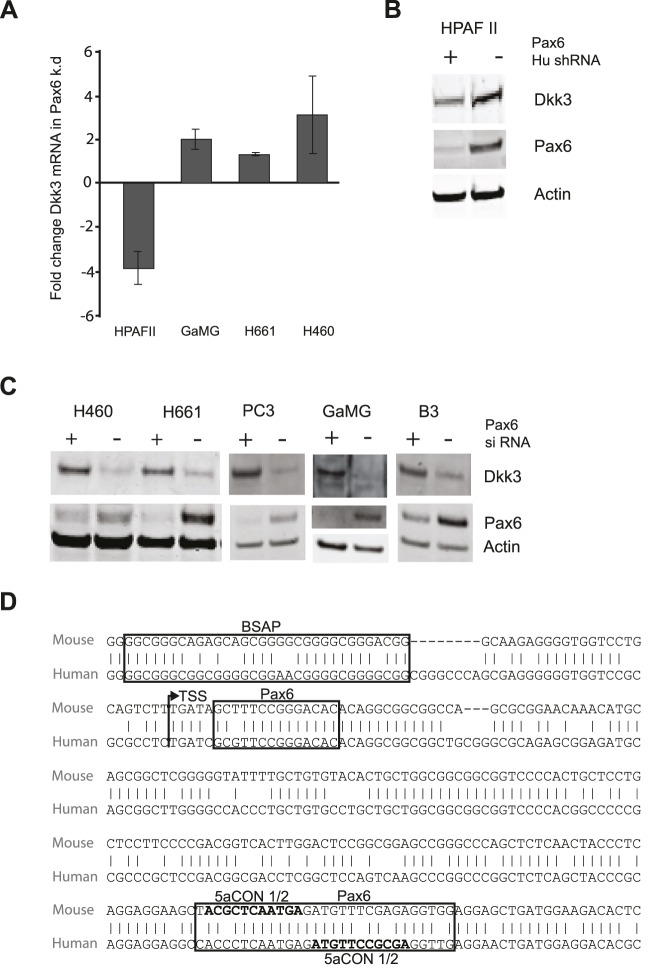
PAX6 regulates the expression of Dkk3 in several human cell lines derived from both normal and tumor tissues. A, RT-qPCR of human cell lines where PAX6 has been knocked down either stably by shRNA (HPAFII), or transiently by siRNA (H460, H661 and GaMG). The result is the mean of 3 independent experiments. B, Western blot of the pancreatic adenocarcinoma cell line HPAFII stably transfected with Pax6 shRNA. C, Western blot of H460 and H661 (lung cancer), PC3 (prostate cancer), GaMG (glioblastoma) and B3 (normal lens epithelium) cell lines with transient knockdown of PAX6 with siRNA. Each western blot in B and C is representative of at least two independent experiments. D, alignment of the human and mouse *Dkk3* promoter showing the localisation of the bioinformatically predicted and evolutionary conserved BSAP (Pax5)-, Pax6- and Pax6(5a) binding sites. The start point for most transcripts in the mouse *Dkk3* promoter is marked by an arrow (TSS-2). The human *Dkk3/REIC* promoter also contains multiple transcripts initiating in this area according to DBTSS. 5aCON½ is a Pax6(5a) consensus halfsite with 2 mismatches.

To see whether Pax6 regulates expression of *Dkk3* in other cell types in addition to the FlpIn-3T3 Pax6- and Pax6(5a) cell lines, several human cancer cell lines were investigated. When *PAX6* was knocked down by stably transfected PAX6 shRNA in pancreatic adenocarcinoma HPAFII cells, a 4-fold decrease in *Dkk3/REIC* gene expression was observed (Fig. 2A). A similar result was obtained by transiently transfecting HPAFII cells with siRNA (data not shown). The expression decrease was confirmed by Western blot (Fig. 2B). However, for the lung cancer cell lines NCI-H661 and NCI-H640, the glioblastoma cell line GaMG and the prostate cancer cell line PC3, PAX6 knockdown by siRNA resulted in an increased Dkk3 expression, indicating that endogenous PAX6 repressed Dkk3 expression in these cancer cell lines. This was also observed for the normal human lens epithelial cell line B3 (Fig. 2A and 2C). PAX6 knockdown did not influence DKK3 expression in the HeLa (human cervix carcinoma) or U-87 (human glioblastoma) cell lines (results not shown). To summarize, this shows that regulation of Dkk3 by PAX6 takes place in several human tissues and cell lines in a context-specific manner, resulting in either up- or downregulation of *Dkk3* gene expression.

### Predicted Pax6 binding sites are conserved between the mouse and human *Dkk3* promoters

To our knowledge there are no publications on transcription factors binding to the *Dkk3* promoter. According to the Database for Transcriptional Startsites (DBTSS) there are two promoter regions in the mouse, and three in the human *Dkk3* gene [36]. The distance between these promoters is approximately 620 bp in mouse and 740 bp (first and last promoter) in humans. In both species the promoters are GC-rich regions with no TATA box present. Transcription starts within a region of 50 bp for each promoter. From here on the two clusters of transcriptional start sites will be called TSS-1 and TSS-2. Alignment of the human- and mouse *Dkk3* promoter shows conservation only around TSS-2, where 77% identicality was observed over a region of 374 basepairs (see alignment in Fig. 2D). When analysed by rVista [39] two evolutionary conserved Pax6 binding sites were identified in this region. Evolutionary conserved binding sites for BSAP (Pax5) were also identified using the ConSite database [40]. Three BSAP sites (two for human) overlapped just 5′ of TSS-2 in the mouse *Dkk3* gene. Since BSAP and Pax6 binding sites’ preferences only differ by 1 nucleotide, and since Pax6 is able to bind the BSAP binding site [41], we considered the identified BSAP sites as possible Pax6 binding sites. The Findpatterns function of the Accelrys GCG software was then used to look for potential Pax6(5a) binding sites [2] in the mouse- and human *Dkk3* promoter (see materials and methods). One site was identified approximately 200 bp downstream of TSS-2 in the mouse *Dkk3* promoter. Interestingly, a 5aCON halfsite was also found in the human *DKK3* promoter, starting just 3′ to the 5aCON halfsite identified in the mouse sequence of this evolutionary conserved region. In each species the 5aCON halfsite is therefore part of an incomplete (not optimal) full 5aCON site. In addition, this site overlaps with the evolutionary conserved Pax6 binding site identified by rVista (Fig. 2D).

### Both Pax6 and Pax6(5a) are capable of activating transcription from a 792 bp cloned fragment of the mouse *Dkk3* promoter

**Figure 3:**
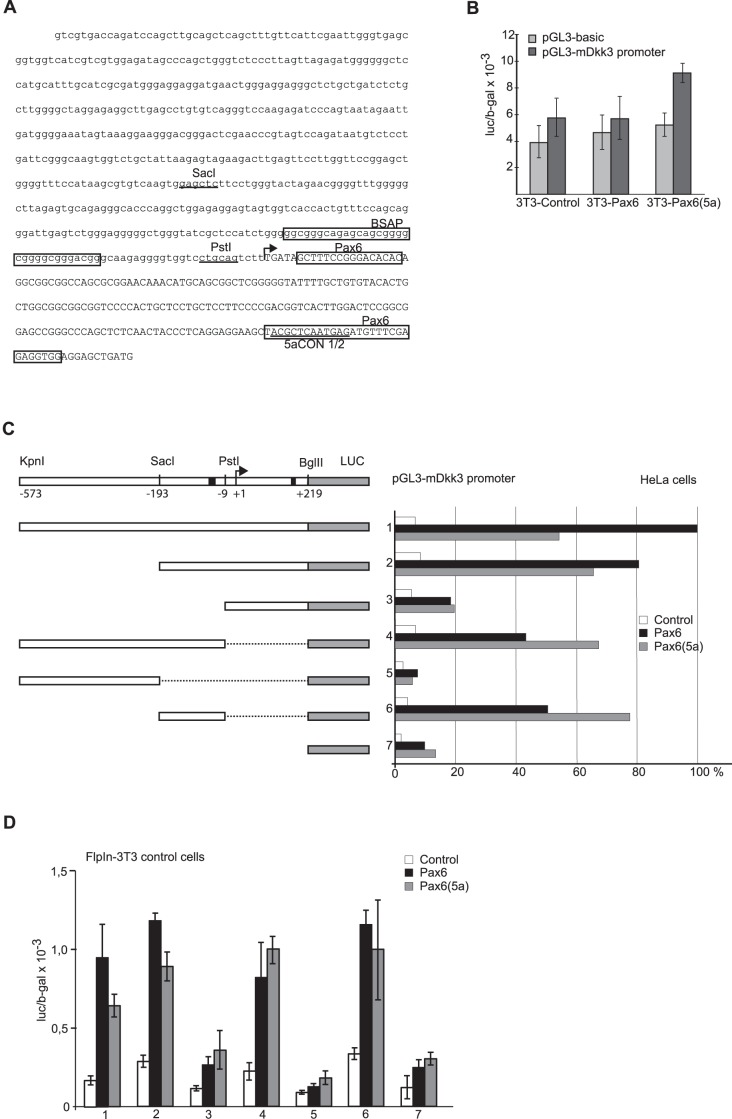
Both Pax6 and Pax6(5a) are potent activators of transcription of the minimal mouse *Dkk3* promoter. A, the nucleotide sequence of the 792*Dkk3* promoter cloned into pGL3-basic. The SacI and PstI restriction enzyme sites used for truncation of the promoter are indicated, along with the bioinformatically predicted binding sites for BSAP (Pax5), Pax6 and Pax6(5a). There are multiple transcriptional start sites within a 40 bp area according to DBTSS. The arrow is placed at the peak of transcriptional initiation in the region called TSS-2. B, transient transfection of a luciferase reporter vector containing the 792 bp fragment(−573/+219) from the mouse *Dkk3* promoter in the FlpIn-3T3 Pax6(5a), -Pax6 and -Control cell lines. The transfections were done in triplicates, and the figure shows one representative experiment of three in total. C, the 792 bp *Dkk3* promoter reporter, 1(−573/+219) and deleted versions of it, 2 (−193/+219), 3 (−9/+219), 4 (−573/−9), 5 (−573/−193), 6 (-193/−9) and 7 (pGL3-basic)., were co-transfected with Pax6 or Pax6(5a) expressing vectors in HeLa cells, identifying the minimal mouse *Dkk3* promoter sequence required for transcriptional activation by Pax6 and Pax6(5a). Transfections were done in triplicates, and co-transfection with pCH110 (β–gal) was used to adjust for transfection efficiency. The figure shows mean values from 4–9 individual transfection experiments done in triplicates. The luc/β–gal value from *Dkk3* promoter construct number 1 co-transfected with Pax6 was set to 100%. The other results from co-transfection experiments with deletion constructs, Pax6 and Pax6(5a) were compared to this. The mean and standard deviations were calculated, and the Student’s t-test showed statistical significance (Fig. S3). D, The transfections done in HeLa cells were repeated in murine FlpIn-3T3 cells using the Lipofectamine 2000 transfection reagent. Transfections were done in triplicates, and co-transfection with pCH110 (β-gal) was used to adjust for transfection efficiency. The figure shows a representative experiment of three in total.

To test if the bioinformatically predicted Pax6 binding sites were functional in transcriptional regulation, we cloned a fragment of the mouse *Dkk3* promoter containing the conserved Pax6 binding sites close to TSS-2. Approximately 600 bp upstream- and 200 bp downstream of TSS-2 were cloned into the pGL3-basic reporter gene vector (Fig. 3A). We first tested the cloned *Dkk3* promoter by transient transfection and reporter gene assays in the three FlpIn-3T3 cell lines. The cloned *Dkk3* promoter showed slightly higher activity in the FlpIn-3T3 Pax6(5a) cells compared to the FlpIn-3T3 control or -Pax6 cells (Fig. 3B), which fits with the RT-qPCR and WB data (Fig. 1A and 1B). However, based on the RT-qPCR results, a bigger difference in *Dkk3* promoter activity was expected. The relatively weak activation of the cloned *Dkk3* promoter in the FlpIn-3T3 Pax6(5a) cells indicated that TSS-1, additional sequences surrounding the TSS-2, or enhancers acting from more distal positions, were required for full transcriptional activity of the *Dkk3* promoter in this cell line.

Further studies of the cloned mouse *Dkk3* promoter were done in HeLa cells. By itself the cloned *Dkk3* promoter fragment had little activity. However, co-transfection with either mouse Pax6- or Pax(5a)-expressing plasmids caused 6–12 fold increase in transcriptional activity, with Pax6 being more efficient than Pax6(5a) in transcriptional activation (Fig. 3C). The evolutionary conserved BSAP- and Pax6/Pax6(5a) binding sites are situated on each side of the TSS-2, with a naturally occurring PstI site in between (Fig. 3A). Deletions from both ends of the promoter by use of the internal SacI and PstI sites gradually decreased the transcriptional activity obtained by co-transfected Pax6 and Pax6(5a) (Fig. 3C). However, a 184 bp region just upstream of the TSS-2 (−193/−9) was still capable of responding to co-transfected Pax6 and Pax6(5a). This region of the *Dkk3* promoter contained the predicted BSAP binding sites. Remarkably, the removal of the 3′ end of the promoter construct (containing the predicted Pax6/Pax6(5a) binding site) decreased the effect of co-transfected Pax6 but not the effect of co-transfected Pax6(5a) (Fig. 3C, compare constructs 1 and 4). While Pax6 was most efficient in activating transcription from the longest *Dkk3* promoter construct, Pax6(5a) caused the highest transcriptional activity from the 184 bp construct (Fig. 3C, compare construct 1 with 4 and 6). Similar results were obtained when the mouse Dkk3 promoter constructs were transfected into the murine FlpIn-3T3 control cell line, indicating that there are no species specific differences in the regulation of the cloned promoter fragment (Fig. 3D).

### EMSA confirms binding of Pax6 to elements in the mouse *Dkk3* promoter

**Figure 4:**
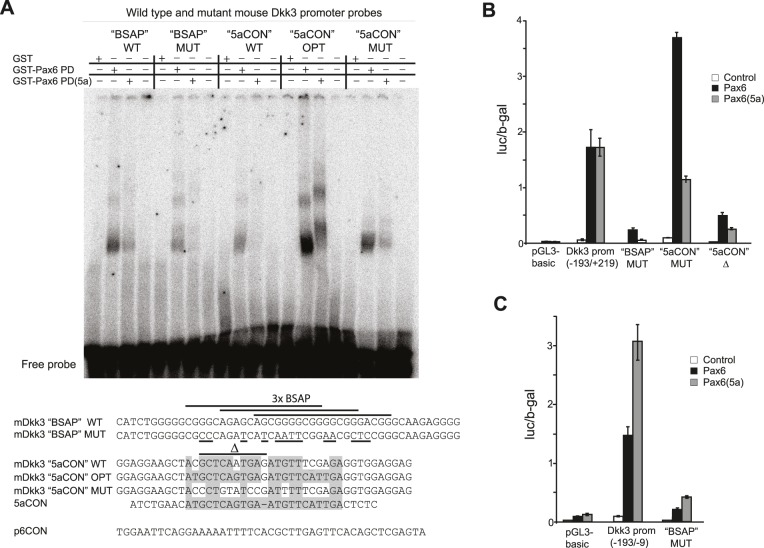
EMSA confirms direct binding by the Pax6 paired domain to an evolutionary conserved binding site in the mouse *Dkk3* promoter, and a mutated version of this site is not longer able to mediate transcriptional activation by Pax6 from the Dkk3 promoter. A, affinity purified recombinant GST-fusion proteins containing the Pax6- or the Pax6(5a) paired domain (PD) were used in EMSA with a 55 bp probe covering the evolutionary conserved BSAP (Pax5) binding site in position −56/−24 at the mouse *Dkk3* promoter. A mutated version of this probe is also included. The “5aCON” WT probe (position +180/+208 according to TSS-2), optimized (OPT) and mutated (MUT) versions of this probe were also used in binding reactions with recombinant Pax6 PD and Pax6(5a) PD. The sequences of the wild type (WT) and mutated (MUT) “BSAP” probes are shown beneath the gel. The exact localization of the three bioinformatically predicted- and overlapping BSAP sites is indicated by three lines above the “BSAP” probe sequence. The nucleotides changed in the “BSAP” MUT sequence are underlined. “5aCON” OPT is an optimized version, similar to the original 5aCON sequence. Grey bars show the homology with 5aCON for each version of the mouse Dkk3 “5aCON” probes. B, transient transfections and reporter gene assays in HeLa cells with pGL3-mDkk3 promoter construct nr 2 (−193/+219) containing the mutated “BSAP” and “5aCON” sites, and a deleted “5aCON” site (“5aCON Δ”). The ten deleted nucleotides in the “5aCON Δ” probe which is replaced with the dinucleotide TT are indicated by a Δ symbol above the sequence in A. C, transient transfections and reporter gene assays in HeLa cells with the minimal 184 bp pGL3-mDkk3 promoter construct, nr 6 (−193/−9) containing the mutated “BSAP” site. Transfections were done as described in figure 3.

To verify whether the predicted BSAP, Pax6 and Pax6(5a) binding sites in the mouse *Dkk3* promoter directly bind Pax6 and Pax6(5a), EMSA was performed. Purified recombinant GST-Pax6 paired domain (PD) proteins (with and without the amino acids encoded by the exon 5a insert) were mixed with a radiolabeled oligonucleotide identical to the 50 bp covering the three overlapping BSAP sites (Fig. 4A). The recombinant Pax6 PD bound the BSAP site well, while a mutated version of this probe was bound less efficiently (Fig. 4A). The recombinant GST-Pax6(5a) PD was not able to bind to the BSAP site probe, which was expected since this probe is not similar to the Pax6(5a) binding site consensus. Puzzling results were obtained with the probe for the predicted Pax6/Pax6(5a) binding site downstream of TSS-2. This probe (named “5aCON” in Fig. 4A) was not bound at all, or bound very weakly, by the recombinant GST-Pax6(5a) PD. However, GST-Pax6 PD was able to bind the “5aCON” probe, but less efficiently than it did with both the optimized (OPT) and mutated (MUT) versions of this probe (Fig. 4A). The result of the EMSA with regard to the “BSAP” probe is in line with the results of the reporter gene assay shown in Fig. 3C, where the predicted BSAP binding site in the *Dkk3* promoter is located in the 184 bp fragment still able to respond to co-transfected Pax6 (construct 6, Fig. 3C). When the “BSAP” binding site mutation was introduced into this minimal 184 bp mDkk3 promoter vector co-transfected Pax6 could no longer activate transcription in HeLa cells (Fig. 4C). The “BSAP” mutation had the same effect when introduced into a slightly larger fragment of the mDkk3 promoter containing both the “BSAP” and “5aCON” binding sites (Fig. 4B). However, when the “5aCON” site was mutated, the transcriptional activation increased (Fig. 4B), which is in line with the EMSA showing stronger binding of Pax6 to the probe when the “5aCON” site was mutated. Importantly, the deletion caused in the “5aCON” binding site by replacement of ten central nucleotides with TT inhibited transcriptional activation by Pax6/Pax6(5a) (Fig. 4B). The co-transfected plasmids used in the reporter gene assays encode full-length Pax6 and Pax6(5a) proteins, while only the recombinant Pax6 PD or Pax6(5a) PD was used in EMSA. To see if the full-length Pax6- and Pax6(5a) proteins had been able to bind the “BSAP” and “5aCON” binding sites, nuclear extracts from the FlpIn-3T3 Control, -Pax6 or -Pax6(5a) cell lines were used in EMSA. Since it turned out that many proteins (also from the FlpIn-3T3 Control cell line) bound these two probes *in vitro* (Fig. S1), we were unable to clarify the contribution of Pax6 and Pax6(5a). Similar to the observed results with the recombinant Pax6 PD, the mutated “BSAP” probe bound less protein, while the mutated or optimized “5aCON” probe bound more protein than the wild type version of the probe. Thus, the 50 bp sequence including the Pax6 binding “BSAP” site just in front of TSS-2 seems to be a hot-spot for protein binding in the mouse *Dkk3* promoter. With that in mind it is remarkable that transcriptional activation from this fragment in reporter gene assays clearly required co-transfected Pax6 or Pax6(5a) (Fig. 3C and Fig. 4C).

### The FlpIn-3T3 Pax6 and -Pax6(5a) cell lines show alterations in the Wnt signaling pathway compared to the control cell line

**Figure 5:**
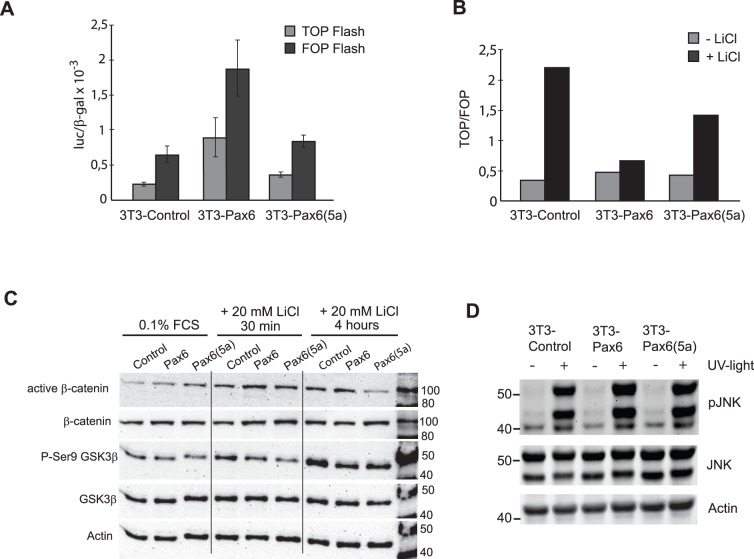
The FlpIn-3T3 Pax6 and Pax6(5a) cell lines display alterations in the Wnt signaling pathway compared to the FlpIn-3T3 control cell line. A, Transient transfections with the TOP and FOP reporter plasmids into the FlpIn-3T3 Control, Pax6 and Pax6(5a) cell lines, showing basal (background) luc/β-gal activities. B, Calculations of TOP/FOP ratios as measures for Wnt signaling pathway response after transient transfections and LiCl stimulation (20 mM) for 20 hours before harvesting of the three FlpIn-3T3 cell lines. C, Western blot of FlpIn-3T3 control, -Pax6 and -Pax6(5a) cell lines serum starved for about 2 days before they were stimulated with LiCl (20 mM) and harvested after 30 minutes and after 4 hours. Antibody against active β-catenin was first used and images were developed. The membrane was then stripped and antibodies against total β-catenin and actin were used. Likewise, an antibody recognizing phosphorylated serine 9 in GSK3-β (inactivated) was first used, before antibodies against total amount of GSK3-β and actin was applied to the same membrane. Results are representative of two biological replicates. D, The ability of the FlpIn-3T3 cell lines to phosphorylate JNK was investigated by Western blot after UV stimulation. Similar results were observed in three biological replicates.

To investigate whether the three generated FlpIn-3T3 cell lines had differences in Wnt pathway activity, we performed a TOPflash/FOPflash transfection and reporter gene assay. The TOP reporter vector contains eight Tcf binding sites, and is responsive to activation of the Wnt pathway, by combined binding of β-catenin and Tcf [34]. In the FOP reporter the Tcf sites are mutated, so this vector should not be responsive to Wnt signaling. However, we observed relatively high activity of both the TOP and the FOP plasmid in the FlpIn-3T3 Pax6 cell line compared to the control- and the FlpIn-3T3 Pax6(5a) cell lines before any stimulation of the Wnt pathway (Fig. 5A). When LiCl was used to activate the Wnt signaling pathway, the resulting normalized TOP/FOP ratios showed that the FlpIn-3T3 Pax6- and Pax6(5a) containing cell lines were 2–3 fold less responsive to the LiCl stimulation compared to the control cell line (Fig. 5B). In principle, this would fit with the theory that the cell lines expressing the highest amount of Dkk3 would be less responsive to Wnt pathway activation. Next we had a look at the levels of activated β-catenin. All three FlpIn-3T3 cell lines were stimulated with 20 mM LiCl 30 minutes and 4 hours before harvesting. Four hours after LiCl stimulation, there was far less active β–catenin in the FlpIn-3T3 Pax6(5a) cell line compared to both the FlpIn-3T3 Control and -Pax6 cell line, while the level of total β–catenin remained the same in all three cell lines (Fig. 5C). It is known that activation of the Wnt pathway causes an inhibitory phosphorylation of Ser 9 in GSK3-β. This inhibition contributes to the stabilization and activation of β–catenin. The Western blot in Fig. 5C clearly showed that there is less GSK3-β Ser 9 phosphorylation in both the FlpIn-3T3 Pax6 and -Pax6(5a) cell lines compared to the control cell line four hours after LiCl stimulation. The level of total GSK3-β was the same in all cell lines. This indicated that the activation of the Wnt pathway was inhibited at the level of GSK3-β in the FlpIn-3T3 Pax6 and -Pax6(5a) cell lines. This experiment was repeated with the human HPAFII 13 (control) and HPAF II 84 (shRNA mediated PAX6 knockdown) cell lines, but we were not able to see consistent changes in the level of activated β-catenin or GSK3-β Ser 9 phosphorylation after 4 hrs of LiCl stimulation (Fig. S2).

Dkk3 is reported to affect apoptosis and JNK signaling [29], and JNK also plays a central role in activation of the non-canonical Wnt pathway [42]. We therefore looked at the JNK expression and phosphorylation in the three different 3T3 cell lines. UV-light was used to induce stress. The results showed that JNK was slightly more expressed in the FlpIn-3T3 Pax6 and -Pax6(5a) cells compared to the FlpIn-3T3 Control cells, and so was the activation measured by phospho-JNK (Fig. 5D). More phosphorylation of JNK in the FlpIn-3T3 Pax6 and -Pax(5a) cell lines probably only reflects the higher amount of JNK protein, and not increased responsiveness to the stress signal.

## Discussion

We have shown that Pax6 regulates the expression of Dkk3 in cell lines derived from mouse and human. Dkk3 is positively regulated by both the Pax6 and the Pax6(5a) isoforms in mouse FlpIn-3T3 cells, and we have identified an evolutionary conserved binding site for Pax6 immediately upstream of TSS-2 in the mouse *Dkk3* promoter. EMSA clearly showed the direct binding of the Pax6 paired domain to this site. Other nuclear proteins were able to bind this site as well, but for transcriptional activation Pax6 binding was essential. Since *Dkk3* belongs to the Dickkopf family of Wnt signaling pathway inhibitors, we investigated whether there were differences in Wnt signaling pathway activity between the FlpIn-3T3 Control cells (low *Dkk3* expression) and the Pax6- and Pax6(5a) expressing FlpIn-3T3 cells (high *Dkk3* expression). Both TOP/FOP reporter gene assay and Western blot of active β–catenin and repressed GSK3-β showed differences between the cell lines, indicating that the canonical Wnt pathway is indeed affected.

There are various reports regarding the methylation status of the *Dkk3* promoter [26], and indications that histone modifications may contribute to regulation of the promoter [29], [30]. However, not much is known about transcription factors and regulatory proteins binding to it. One study reports that p53 is responsible for the increase in *Dkk3* expression observed after knockdown of Cdc7 and replication checkpoint arrest in a fibroblast cell line [43]. These observed effects are not associated with a particular binding site or region of the *Dkk3* promoter. In addition there are two recent studies showing that *Dkk3* expression is regulated by miRNA-92 in neuroblastoma cell lines [27], [28]. Lately it was shown that Pax6 and Mitf enabled Wnt signaling in the retina pigmented epitelial cells by repressing *Dkk3* and *Fgf19* [44]. A Pax6 binding site was identified in a distal enhancer, and this mediated weak repression of the minimal *Dkk3* promoter in the ARPE19 (human retinal pigmented epithelial) cell line. In that study the activity of the minimal promoter in combination with Pax6 was not shown, so the results can therefore not be directly compared to our findings.

Our results show that the regulation of *Dkk3* by PAX6 takes place in several cell lines from mouse and human. Since *Dkk3* and *Pax6* are expressed in overlapping areas in both the brain and the eye during embryo development [45], [46], there is a possibility for *Dkk3* expression to be regulated by Pax6 *in vivo* in several tissues. In support of this, *Dkk3* was one of the downregulated genes in Pax6 deficient telencephalon, indicating that Pax6 normally upregulates *Dkk3* expression in this tissue [47]. It seems to be cell/tissue specific whether Pax6 activates or represses *Dkk3* expression, and this is in line with what we observed in our experiments. The fact that a gene can be regulated in opposite directions by Pax6 in different tissue is also observed by others [10].

While other members of the Dickkopf family (Dkk1, Dkk2 and Dkk4) have been shown to bind LRP5/6 and function as Wnt pathway inhibitors [19], the function of Dkk3 is somewhat more enigmatic. Dkk3’s interaction with Krm takes place on intracellular membranes located in the perinuclear region (possibly ER) in HEK293 and SH-SY5Y cells, and in these cells Dkk3 leads to activation, not inhibition, of Wnt signaling [20]. This observation was recently confirmed in the reprogramming of embryonic lung fibroblasts to smooth muscle cells where Dkk3 plays a central role [48]. Contradictory to this, Dkk3 is reported to function as an inhibitor of Wnt signaling, either by associating with bTrCP and blocking β–catenin from entering the nucleus (as seen in HeLa cells) [38], or by increasing the localisation of β–catenin in the cell membrane, as seen in osteosarcoma cells [21]. We found less active β-catenin and observed less TOP/FOP reporter gene activity in the FlpIn-3T3 Pax6- and Pax6(5a) cell lines (compared to the control cell line) after stimulation of the Wnt pathway with LiCl. This supports the idea that Dkk3 in the 3T3-fibroblast cell line functions as an inhibitor of the Wnt pathway. However, since we (this work) and others [44], [47] have shown that Pax6 can enhance or repress *Dkk3* expression, and since Dkk3 can enhance or repress Wnt signaling through the canonical pathway depending on the cell type, it will be important for future studies to reveal whether there is a correlation between Pax6 regulation of Dkk3 and the net effect this has on the Wnt pathway.

There are several reports on interactions between Pax6 and the Wnt pathway. Pax6 regulates the expression of several Wnt pathway components, both in the brain [49] and in the eye [15]. It is also known that the Wnt signaling pathway is able to influence Pax6 expression [50], [51]. Since the Wnt pathway inhibitors Sfrp1 and Dkk1 are upregulated by Pax6 [15], it is possible that the observed inhibition of Wnt pathway activation by LiCl in the Pax6- and Pax6(5a) containing 3T3 cell lines can be caused by factors other than Dkk3. Nevertheless, we have shown that the presence of Pax6 and Pax6(5a) in FlpIn-3T3 cells makes these cells less responsive to activation of the Wnt pathway.

## Materials and Methods

### Cell culture

FlpIn-3T3 cell lines (R761–07, Invitrogen, Life Technologies, Carlsbad, CA) were grown in Dulbecco’s Modified Eagles Medium (DMEM) - high glucose (Sigma-Aldrich, St.Louis, MO) supplemented with 10% fetal calf serum (FCS) (Biochrom AG, Berlin, Germany) supplemented with the appropriate antibiotics, see [9] for construction of the cell lines. The human pancreatic adenocarcinoma cell line HPAFII (ATCC CRL-1997, Rockville, MD) was cultured in Eagle’s minimum essential medium (MEM) (Sigma-Aldrich) with 10% FCS (Biochrom AG, Berlin, Germany). The human glioblastoma cell lines GaMG [31], U-251 MG (cat#09063001, Sigma-Adrich) and U-87 MG (ATCC HTB-14) were grown in Dulbecco’s Modified Eagles Medium - high glucose (Sigma-Aldrich) with 10% FCS (Biochrom AG). PC3 human prostate cancer cells (ATCC CRL-1435) were cultured in F-12K medium (cat#211-27, Gibco, Life Technologies, Carlsbad, CA) with 10% FCS. Human large cell lung carcinoma cell lines NCI-H661 (ATCC HTB-183) and NCI-H460 (ATCC HTP-177) were grown in RPMI-1640 (Sigma-Aldrich) with 10% FCS. The human lens epithelial cell line B3 (ATCC CRL-11421) was grown in MEM supplemented with 2 mM L-glutamine, 0.1 mM non-essential amino acids, 1 mM Sodium-puryvate and 20% FCS, while human cervical carcinoma cell line HeLa (ATCC CCL2) was grown in MEM supplemented with 2 mM L-glutamine, non-essential amino acids and 10% FCS. Media for all cell lines contained 1% of mixed 100 U/ml penicillin and 100 mg/ml streptomycin (cat#P0781, Sigma-Aldrich).

### Stimulation of cells with UV-light and LiCl

Cells seeded in 6-well plates were UV-stimulated for 15 minutes using a single UV benchtop transilluminator (UVP, Upland, CA), harvested by washing with PBS, adding 100 µl 2x SDS per well, scraped, transferred to 1,5 ml tubes and immediately heated at 100°C for 5–10 minutes. For LiCl stimulation, cells were serum starved in 6-well dishes for approximately 48 hours before LiCl was added to a final concentration of 20 mM. Cells were harvested after 30 minutes and after 4 hours as described above.

### RNA interference

Cells were transfected in 6-well plates with human PAX6 siRNA (#114168 Silencer Select PAX6 siRNA, Ambion) using Lipofectamine 2000 (#11668-027, Invitrogen). A scrambled negative control siRNA was included in all experiments (Silencer Negative Control #2 siRNA, Ambion). Cells were harvested 48 hrs after siRNA transfection.

29 mer HuSH shRNA constructs were purchased from Origene (TG310597, Rockville, MD). Lipofectamine 2000 was used to transfect the HPAFII cell line with either a construct targeting Pax6 (84A) or a non-silencing control construct (13A). Cells with stably integrated shRNA constructs were selected in growth medium containing 1 µg/ml puromycin (Sigma-Aldrich) for several passages. Knockdown of Pax6 in HPAFII 84A was verified by Western blot and RT-qPCR.

### RNA extraction and RT-qPCR

Total RNA was extracted using the RNeasy Plus kit (#74134, Qiagen, Hilden, Germany). Reverse transcription of total RNA was performed with Superscript III Reverse Transcriptase kit (#18080-044, Invitrogen), using 150 ng random hexamer primers (Fermentas International Inc., Canada). dNTP mix was purchased from Promega (Madison, WI). We used 500 ng total RNA per 20 µl cDNA reaction. Primer pairs were designed using Primer 3 software (Whitehead Institute, Cambridge, MA) and synthesized by Invitrogen or Sigma, or purchased directly from Qiagen (table 1 and 2). For quantification of mRNA expression levels, a Stratagene MX3000P instrument (Stratagene, La Jolla, CA) was used. cDNA corresponding to 25 ng RNA was amplified for 40 cycles in a 25 µl SYBR green PCR mix (Brilliant II SYBR Green QPCR master mix, Stratagene) containing 200 nM of each primer. Cycling conditions were as follows: 95°C for 10 min followed by 40 cycles at 95°C for 30 sec, 60°C for 1 min and 72°C for 30 sec. Duplicate PCR analyses were performed on each cDNA sample. The absence of genomic DNA and contaminations were confirmed by the inclusion of no reverse transcriptase (No RT) controls and no template controls (NTCs) respectively. The relative amount of target gene normalized to the average expression of the two human reference genes *GUSB* and *TFRC,* or the two mouse reference genes *Nono* and *Tfrc,* was determined using the ΔΔCq-method [32].

**Table 1:** Primers for RT-qPCR.

Gene symbol	Forward primer (5′-3′) RT5	Reverse primer (5′-3′) RT3	Amplicon length (bp)	RefSeq Accession Number
*hDKK3*	CTGGGAGCTAGAGCCTGATG	TCATACTCATCGGGGACCTC	168	NM_015881.5 NM_013253.4 NM_001018057.1
*hPAX6*	CAACTCCATCAGTTCCAACG	TGGATAATGGGTTCTCTCAAACTCT	145	NM_001604.4 NM_000280.3 NM_001127612.1
*mDkk3*	GCAGCTGCTAAAACGTCCTC	GACCACCTGTCCACTCTGGT	153	NM_015814.2
*mNono*	TGGAAAAGCAGGCGAAGTTT	TTTCCGCTAGGGTTCGTGTT	80	NM_023144.2 NM_001252518.1

Abbreviations: *h*; human, *m*; mouse, *DKK3*; Dickkopf 3, *Nono*; Non-POU-domain-containing octamer binding protein.

**Table 2:** Qiagen Quantitect primer assays.

Gene symbol	Assay name	Cat.No	Amplicon length (bp)	RefSeq Accession Number
*hTFRC*	Hs_TFRC_1_SG	QT00094850	81	NM_001128148 NM_003234
*hGUSB*	Hs_GUSB_1_SG	QT00046046	96	NM_000181
*mTfrc*	Mm_Tfrc_1_SG	QT00122745	106	NM_011638

Abbreviation: *h;* human, *m;* mouse *TFRC*; Transferrin receptor, *GUSB*; Glucuronidase beta.

### Preparation of nuclear extracts for Western blot

Cells were grown to 70–80% confluency in two 175 cm^2^ bottles, trypsinized and harvested. After washing with PBS cells were incubated with one packed cell volume (1 pcv) buffer A (10 mM Tris HCl (pH 8), 10 mM KCl, 1.5 mM MgCl_2_, 1 mM DTT) containing protease inhibitors (cat#11 836 153 001, Roche Diagnostics, Mannheim, Germany) for 15 min on ice. Cells were crushed by 8 rapid strokes using a 1 ml syringe with a 25 g needle and centrifuged for 20 sec at >13000×g (4°C). The supernatant was transferred to a separate tube (cytoplasm), while the pellet containing the nuclei was resuspended in 2/3 pcv of buffer C (50 mM Tris HCl (pH 8), 0.42 M KCl, 5 mM MgCl_2_, 0.1 mM EDTA, 20% Glycerol, 10% Sucrose, 1 mM DTT) with protease inhibitors, and cooled at 4°C for 30 min before centrifugation for 5 min at >13000×g (4°C). The supernatant containing nuclear extract was transferred to a separate tube. The nuclear pellet (the insoluble remnants) was saved for later analyses. The BioRad reagent (#500-0006, BioRad Laboratories) was used to determine the protein concentration. Fifteen µg were loaded on a SDS-PAGE gel for western blot. The nuclear pellet was resuspended and boiled in 2x SDS gel loading buffer, and 1/5 of the volume was put on the gel.

### Western blot

Prepared protein samples were run on a 10% SDS-PAGE gel (Mighty Small, Invitrogen), and blotted onto a Hybond nitrocellulose membrane (GE Healthcare) using the Mighty Small blotting system (Invitrogen). The membrane was incubated with blocking buffer (PBS buffer/0.1% Tween-20/5% non-fat dried milk). Alternatively, samples were run on NuPAGE Novex 4–12% Bis-Tris gels (Invitrogen), and blotted onto the Odyssey nitrocellulose membrane (LI-COR Biosciences, Lincoln, NE). Membranes were blocked using Odyssey blocking buffer (LI-COR Biosciences). Primary and secondary antibodies were diluted in the blocking buffer (20 µl Tween-20 added per 5 ml of the Odyssey blocking buffer), and membranes were incubated over night at 4°C, or at room temperature for at least 1 hour. Washing was done 5 times for 5 minutes in PBS or TBS containing 0.1% Tween-20 after each antibody incubation. Hybond membranes were incubated with HRP-conjugated secondary antibodies, and developed using the Western Blotting Luminol Reagent (sc-2048, Santa Cruz, Dallas, TX). Images were acquired on an Image Reader LAS-3000 Fujifilm. The LI-COR membranes were incubated with IRDye Coupled secondary antibodies, and images were acquired with the Odyssey Sa Infrared Imaging System (LI-COR Biosciences).

Antibodies used were rabbit anti-Pax6 antibody 1∶1200 (AB2237, Merck Millipore, Billerica, MA,), rabbit anti-Actin 1∶2000 (A2066, Sigma-Aldrich), goat anti-Dkk3 1∶4000 (ab2459, Abcam), rabbit anti-phospho-JNK 1∶1000 (#9251, Cell Signaling Technology, Beverly, MA), rabbit anti-JNK 1∶1000 (#9252, Cell Signaling Technology), mouse anti-active β-catenin 1∶2500 (#05-665, Merck Millipore), rabbit anti-β-catenin 1∶4000 (ab6302, Abcam), anti-GSK3β-Ser9 1∶1000 (#9322, Cell Signaling Technology), anti-GSK3β-total 1∶500 (sc-9166, Santa Cruz Biotechnology), anti-rabbit HRP-conjugate 1∶2000 (#554021, BD Biosciences, San Jose, CA), anti-biotin HRP-linked antibody 1∶2000 (#7075, Cell Signaling Technology). IRDye secondary antibodies: anti-goat 800 CW 1∶10 000 or anti-rabbit 800 CW 1∶10 000 (#926-32214 and #926-32213, LI-COR Biosciences). Molecular weight markers used were Prestained Protein marker, Broad range (#77077S NEB, Ipswich, MA), Biotinylated protein marker (#7727, Cell Signaling Technology), SeeBlue Plus2 Prestained Standard (Invitrogen) and MagicMark XP Western Protein Standard (Invitrogen).

### Construction of reporter gene plasmids containing the Dkk3 promoter

The mouse *Dkk3* promoter was amplified by PCR from 3T3 fibroblast genomic DNA using primers 5′-AAAGGTACCGTCGTGACCAGATCCAGCTT-3′(KpnI) and 5′-AAAAGATCTCATCAGCTCCTCCACCTCTC-3′(BglII). The PCR product (792 bp) was first cloned into pCR-TOPO vector (Invitrogen), confirmed by sequencing, and then subcloned utilizing the restriction sites KpnI and BglII to the pGL3-basic vector ( = pGL3-mDkk3prom). This is construct number 1 containing 573 bp upstream, and 219 bp downstream of the predicted TSS (−573/+219). Deletions from the 5′ and 3′ end of this promoter construct were done using the internal SacI and PstI restriction sites in combination with either KpnI or BglII, followed by agarose gel purification and self-ligation of the truncated pGL3-mDkk3prom vector (construct 2–6 in Fig. 3C). The content of each construct relative to the TSS is as follows: 2 (−193/+219), 3 (−9/+219), 4 (−573/−9), 5 (−573/−193) and 6 (−193/−9). All constructs were verified by sequencing.

### *In vitro* mutagenesis

The pGL3 plasmid containing mDkk3 promoter construct nr 2 was used as template to mutate the “BSAP” site, and to mutate and delete the “5aCON” site. In addition, the “BSAP” site mutation was also introduced into the pGL3 plasmid containing mDkk3 promoter construct nr 6. *In vitro* mutagenesis reactions were performed by the use of primers containing the specific mutations (see Table 3) in PCR reactions with Pfu Ultra High-Fidelity DNA polymerase (Agilent Technologies) according to the manufacturs instruction. PCR products were treated for one hour with DpnI at 37°C before they were transformed into competent *E.coli* DH5a cells. All mutations were verified by sequencing.

**Table 3:** Oligonucleotides used in EMSA and *in vitro* mutagenesis.^#^

mDkk3 “BSAP” WT	CATCTGGGGGCGGGCAGAGCAGCGGGGCGGGGCGGGACGGGCAAGAGGGG
mDkk3 “BSAP” MUT	CATCTGGGGGCGCCCAGATCATCAATTCGGAACGCTCCGGGCAAGAGGGG
mDkk3 “5aCON” WT	GGAGGAAGCTACGCTCAATGAGATGTTTCGAGAGGTGGAGGAG
mDkk3 “5aCON” OPT	GGAGGAAGCTATGCTCAGTGAGATGTTCATTGAGGTGGAGGAG
mDkk3 “5aCON” MUT	GGAGGAAGCTACCCTGTATCCGATTTTTCGAGAGGTGGAGGAG
mDkk3 “5aCON” DEL	CCCTCAGGAGGAAGCTACTTATGTTTCGAGAGGTGGAGG
5aCON	ATCTGAACATGCTCAGTGAATGTTCATTGACTCTC
P6CON	TGGAATTCAGGAAAAATTTTCACGCTTGAGTTCACAGCTCGAGTA

#The reverse complement of each oligonucleotide was also ordered.

### Reporter gene assay

HeLa cells were seeded at a density of 6×10^4^ cells per well in a 6-well dish the day before transfection with Lipofectamine 2000. Each well contained 0.4 µg of the pKW-Pax6(5a), pKW-Pax6 [33], or the empty plasmid pKW10, in combination with 0.4 µg reporter plasmid (pGL3-mDkk3promoter, construct 1–6) and 0.05 µg of the pCH110 plasmid (expressing β-galactosidase (β-gal) for normalization of transfection efficiency). Harvesting was done after 48 hours, and the Dual-Light System (T1003, Applied Biosystems) was used for measurements of luciferase (luc) and β-gal values on a Luminoskan RT dual injection luminometer (Labsystems). All transfections were done in triplicates and repeated 4–9 times. The mean luc/β-gal value for pKW-Pax6 in combination with pGL3-mDkk3promoter (construct nr 1) was set to 100% in each transfection, and then the luc/β-gal values obtained for all the other constructs included in the same transfection were related to this. For reporter gene assay transfection in FlpIn-3T3 control cells the cells were seeded in 24 well dishes the day before transfection, and a total of 1,05 µg DNA (0,5 µg reporter, 0,5 µg effector and 0.05 µg pCH110) and 2 µl Lipofectamine was used per well. For TOP/FOP Flash assays FlpIn-3T3 Pax6, -Pax6(5a) and -control cells were seeded in 6-well plates and transfected with 0.5 µg M50 Super 8×TOPflash or M50 Super 8×FOPflash reporter plasmids per well (Addgene plasmids 12456 and 12457, [34]) together with 0.05 µg pCH110 per well, using Lipofectamine 2000. 20 mM LiCl was added 24 hours after transfection, and harvesting was done after 24 hours as described above. All transfections were done in triplicates and repeated at least 3 times.

### EMSA

The recombinant GST-Pax6 fusion proteins containing either paired domain (PD), or paired domain with 14 amino acid insert (PD5a) were used for protein-DNA interaction studies. GST-fusion proteins were purified from *Escherichia coli* LE392 extracts using glutathione-sepharose beads (#17-5132-01, Amersham Biosciences, Little Chalfont, UK), and were eluted with 20 mM reduced glutathion (G4251, Sigma-Aldrich). For probe generation, 6,25 ng of annealed probes were γ^32^P^–^ATP (Perkin Elmer) labeled with T4 polynucleotide kinase (#2021A, Takara Bio Inc, Tokyo, Japan). Probe sequences are given in Table 3. Labeled probes were purified using QIAquick Nucleotid Removal Kit (#28304, Qiagen). The binding reaction was done essentially as described in [35]. One microgram Poly(dI-dC) (#27-7880, Amersham Biosciences) was used as nonspecific competitor per binding reaction. Two µg of purified recombinant proteins or 10 µg of nuclear extracts were incubated with 20000 cpm probe for each EMSA reaction. DNA-protein complexes were resolved on a 5% TBE-based polyacrylamide gel. Gels were dried and exposed on imaging plates overnight, and scanned at FUJI BAS-5000 analyzer.

### Bioinformatic analyses

Identification of Pax6- and Pax6(5a) binding sites was done as described in [9]. The database for transcriptional start sites (DBTSS) [36] was used to identify transcriptional start sites in the human and mouse *Dkk3* genes.

### Immunocytochemistry and confocal imaging

3T3 cells were seeded at a concentration of 2.4×10^4^ cells per well into an 8-well chambered coverglass (#155411, Thermo Scientific, Rochester, NY, US). The next day cells were fixed in 4% paraformaldehyde for 10 min on ice, permeabilized with ice-cold methanol for 10 minutes on ice, and washed twice with PBS. The cells were blocked in PBS with 3% goat or donkey serum for 60 min in room temperature. Cells were incubated with rabbit anti-PAX6 antibody (AB2237, Merck Millipore), diluted 1∶800 in PBS with 1% goat serum, or goat anti-DKK3 antibody (ab2459, Abcam), diluted 1∶1000 in PBS with 1% donkey serum, for 30 minutes in RT. Cells were washed six times, incubated with Alexa Fluor 488 goat anti-rabbit IgG, diluted 1∶500 in PBS with 1% goat serum (A-11008, Invitrogen) or Alexa Fluor 488 donkey anti-goat IgG, diluted 1∶500 in PBS with 1% donkey serum (A-11055) for 30 min in the dark. Cells were then washed four times, and incubated for 10 minutes with DRAQ5 (1∶1000 in PBS, DR50200, Bio Status Limited, Leicestershire, UK). Cells were washed four times and analyzed in the microscope. Images were acquired with a Zeiss LSM 510 META confocal microscope and the ZEN microscope software.

## Supporting Information

Figure S1 The evolutionary conserved Pax6 (“BSAP” and “5aCON”) binding sites in the Dkk3 promoter are bound by several nuclear proteins. Ten micrograms nuclear extracts from FlpIn-3T3 Control, -Pax6 and -Pax6(5a) cell lines were used in EMSA with the wild type and mutated versions of the “BSAP” and “5aCON” binding sites. The original P6CON [6], and 5aCON [2] probes are *in vitro* selected Pax6 and Pax6(5a) binding sequences, and these were used as positive controls in the panel to the right. The sequences of the probes are shown in Fig. 4.
(EPS)

Figure S2 HPAF II cells with normal and knocked down expression of PAX6 show no consistent difference in Wnt pathway activity upon LiCl stimulation. HPAF 13 (control) and HPAF 84 (PAX6 knocked down) cells were serum starved for 24 hours and then stimulated with 20 mM LiCl for 4 hours before harvesting. Western blotting with antibodies against active β-catenin, total β-catenin, phosphorylated serine 9 of GSK3-β, total GSK3-β and actin was done as described in Fig. 5C. Experiments 1–3 are three biological replicates.
(EPS)

Figure S3 Statistical significance of the results obtained for the transient transfection and reporter gene assays done in HeLa cells with the various mDkk3 promoter fragments co-transfected with Pax6 and Pax6(5a). The 792 bp *Dkk3* promoter reporter and deleted versions of it, were co-transfected with Pax6 or Pax6(5a) expressing vectors in HeLa cells, identifying the minimal mouse *Dkk3* promoter sequence required for transcriptional activation by Pax6 and Pax6(5a). Transfections were done in triplicates, and co-transfection with pCH110 (β-gal) was used to adjust for transfection efficiency. There were no differences in the overall trend of which constructs did respond to Pax6/Pax6(5a) co-transfection, and which did not, but the level of the response and if it was Pax6 or Pax6(5a) that gave the best response varied. In this case it was not possible to choose one experiment as “representative”, so we increased the number of transfections and combined the results of independent experiments. To be able to do this we set the value obtained for the pGL3-mDkk3 promoter (construct nr 1) co-transfected with Pax6 to 100% in each experiment, and compared the result of all other transfections in that experiment to this. Next, the mean percent values for all transfected construct were calculated and presented as shown in Figure 3C. The Student’s t-test (done in Excel, two-sided, assuming unequal variance) showed significant differences for all but two of the transfections when compared to the one given the 100% value. (p<0.01, for 16 of the transfections, p<0.05 for two). The two transfection which was not significantly different (#) had values both higher and lower than 100% in a total of 7 and 8 different transfection experiments. The figure shows mean values and standard deviations from 4–9 independent experiments done in triplicates for each construct.
(EPS)
